# No significant post-operative limb length difference following medial opening wedge high tibial osteotomy in a multi-ethnic Southeast Asian population

**DOI:** 10.1186/s40634-023-00654-4

**Published:** 2023-08-30

**Authors:** Brjan Kaiji Betzler, Sandip Singh Saggi, Matthias Yi Quan Liau, Don Thong Siang Koh, Kong Hwee Lee, Hamid Rahmatullah Bin Abd Razak

**Affiliations:** 1 Lee Kong Chian School of Medicine, Nanyang Technological University, 59 Nanyang Drive, Experimental Medicine Building, Singapore, 636921 Singapore; 2 Department of Orthopaedic Surgery, Sengkang General Hospital, 110 Sengkang East Way, Singapore, 544886 Singapore; 3 Department of Orthopaedic Surgery, Singapore General Hospital, 31 Third Hospital Ave, Singapore, 168753 Singapore; 4 SingHealth Duke-NUS Musculoskeletal Sciences Academic Clinical Programme, Academia Level 4, 20 College Road, Singapore, 169865 Singapore

**Keywords:** Osteoarthritis, High tibial osteotomy, Opening wedge, Limb length, Knee

## Abstract

**Purpose:**

This study aims to assess the significance of post-operative change in limb length in medial opening wedge high tibial osteotomy (MOWHTO), and evaluate the correlation between correction angles and changes in limb length. We hypothesize that opening wedge height and correction angles directly correlate to changes in limb length.

**Methods:**

The medical records and radiographs of 91 MOWHTO patients were retrospectively evaluated for relevant radiographical parameters both pre- and post-operatively. The exclusion criteria are: (i) concurrent distal femur osteotomy in the same setting, (ii) other previous unilateral lower limb surgeries prior to MOWHTO and (iii) failure to follow-up with post-operative radiographs. A linear regression model was performed and a line of best fit, Pearson's correlation coefficient (r) and coefficient of determination (*R*^2^) were computed. Chi-squared test was also performed, and results with *P* < 0.05 were considered statistically significant.

**Results:**

There is a statistically significant increase in post-MOWHTO limb length (Absolute **Δ** = 4.3 ± 2.86 mm, % **Δ = **0.652% ± 0.434%, *p* < 0.001). There was no significant difference in the limb length change between patients with larger (> 8 mm) and smaller (< 8 mm) opening widths.

There was a weakly positive correlation between limb length change versus actual (*R*^2^ = 0.140, 95%CI [-0.068, 0.336]) and planned correction angles (*R*^2^ = 0.196, 95%CI [-0.012, 0.387]).

**Conclusion:**

In conclusion, post-MOWHTO change in limb length is statistically significant, but the clinical significance is minimal. Further studies are required to assess other factors. Development of a reliable mathematical model that predicts post-MOWHTO limb length change would be useful in predicting the anatomical outcomes.

**Level of evidence:**

Level III. Retrospective Cohort Study.

## Introduction

Medial opening wedge high tibial osteotomy (MOWHTO) is a well-reported procedure in the management of patients with medial compartment osteoarthritis with varus deformity [3]. In these patients, the mechanical axis of the lower limb passes through the arthritic medial compartment, contributing to pain and decreased function [16]. MOWHTO seeks to correct this malalignment via the creation of an opening wedge in the proximal tibia, angling the distal tibia in a valgus direction.

Limb length discrepancy (LLD) results in numerous biomechanical and functional complications, including osteoarthritis, scoliosis, and gait instability [21]. This is due to tremendous levels of uneven stress in the back and lower limbs [4]. In theory, MOWHTO has the potential to cause a change in length of the involved leg due to the presence of an opening width. A recent meta-analysis published by Lee *et al. *[15] highlighted concerns in MOWHTO patients who experienced clinical symptoms as a result of leg length change. A significant LLD would be counterproductive to the intentions of MOWHTO predisposes the patient to further biomechanical complications. In a literature search, there seems to be a lack of a definitive consensus on where the threshold lies for a clinically significant LLD. However, Hinarejos *et al. *and Khamis et al. currently report an estimated clinically significant LLD that lies at 10 mm [9, 11]. Conversely, some outlying manuscripts in the literature suggest that LLD > 20 mm can still be compensated [19]. This is important as it proposes that statistical significance may not correlate completely with clinical significance.

Knowing the correlation between wedge height and change in limb length would be useful for surgeons in planning of the MOWHTO as well as for pre-operative counselling of patients prior to MOWHTO if a larger or clinically significant LLD is to be expected post-operatively.

We hypothesize that opening wedge height and correction angles have a direct correlation with changes in limb length in HTO patients.

The aim of the study is to retrospectively assess the significance of post-operative change in limb length following MOWHTO and evaluate the correlation between correction angles and changes in limb length. Creation of a reliable mathematical model that predicts post-MOWHTO limb length change would be beneficial to surgeons in predicting the anatomical outcomes of HTO.

## Methods

The study comprised a total of 91 patients who underwent MOWHTO by two fellowship-trained surgeons working in tertiary teaching hospitals from 2019 to 2022. Patients’ medical records and radiographs were retrospectively reviewed by two independent reviewers. The inclusion criterias were (i) consecutive patients with medial compartment osteoarthritis with varus osteoarthritis who underwent MOWHTO by the two fellowship-trained surgeons, and (ii) availability of pre- and post-operative long leg radiographs. Patients of only these two surgeons were included as they underwent the same fellowship and performed the HTO procedure in the same manner. The surgical technique has been described in detail in a prior publication [10]. The exclusion criteria was: (i) concurrent distal femur osteotomy in the same setting, (ii) other previous unilateral lower limb surgeries prior to HTO and (iii) unavailability of pre- and post-op long leg films. This study was approved by our central institutional review board (CIRB 2022/2115).

The primary outcome of the study was to investigate the statistical significance of limb length change in MOWHTO patients. The secondary outcomes were to investigate the correlation between planned and actual correction angle versus limb length change. Power calculation was performed to estimate the effect size. As per Lee et al.[15], the anticipated mean of the known population was 6.96 mm ± 8.95 mm, with an estimated mean of our study group as 4 mm. Type I error rate was fixed at 0.05, with a power of 80% (β = 0.2). Based on the above, the calculated effect size was 72.

The data collected from imaging consisted of pre- and post-operative tibial lengths, femoral lengths, total limb lengths, hip-knee-ankle angles (HKA), medial proximal tibial angles (MPTA), and posterior tibial slopes (PTS). Tibial length was defined as the distance between the lowest point of the intercondylar tuberosity and the centre of the ankle joint. Femur length was defined as the distance between the centre of the femoral head to the intercondylar notch. Limb length was defined as the distance between the centre of the femoral head to the centre of the ankle joint.

The HKA was defined as the angle between a line from the centre of the femoral head to the femoral intercondylar notch, and a second line from the tibial interspinous point to the middle of the ankle [7]. MPTA was defined as the angle between the tibial mechanical axis and the proximal articular surface of the tibia in the coronal plane [18]. PTS was defined as the angle between the longitudinal axis of the tibia and the posterior inclination of the tibial plateau [2].

The planned correction angle, actual correction angle, and opening width were obtained from pre-operative digital and manual planning records as well as intra-operative notes. Patient demographics, including age, gender, ethnicity, and pre-operative height, weight, and body mass index (BMI) were also collected.

Given two observers, the intra- and interrater reliability were measured using two-way mixed average-measure intraclass correlation coefficient (ICCs), with expected reliability of 0.8 and precision of  ± 95% confidence intervals (CIs). A reliability of more than 0.8 is considered excellent; 0.6–0.8 is good; 0.4–0.6 is moderate and  < 0.4 is considered poor correlation [14]. The calculated sample size was 14 patients, and we had recruited 25 patients for our reliability testing.

SPSS 28 software was used for statistical analysis. The data was plotted onto a graph with a comparative axis of height of osteotomy vs change in limb length. Pearson's correlation coefficient (r) and coefficient of determination (*R*^2^) [6] were computed to assess the correlation between parameters. Comparisons between pre- and post-operative values, as well as planned versus actual correction angle were performed by using the Wilcoxon signed-rank test [17]. Results with *P* < 0.05 were considered statistically significant.

## Results

The mean age of the HTO patients was 54.8 ± 8.3 years. Of the 91 HTO patients, 41 were males and 50 were females, with a good distribution of gender, laterality and ethnicity. The demographics of the HTO patients are shown in Table 1.

**Table 1:** Patient demographics

Age	54.8 ± 8.3
Gender	
Male	41
Female	50
Laterality	
Left	38
Right	53
Ethnicity	
Chinese	60
Malay	18
Indian	11
Others	2

Table 2 shows the pre-operative and post-operative limb lengths, tibial lengths, femoral lengths, HKA angles, MPTA, PTS, planned and actual correction angles (Table 2). The primary outcome of the study was to investigate the statistical significance of limb length change in OWHTO patients. There was a statistically significant increase in the post-HTO limb length from 541.2 ± 34.9 mm to 545.5 ± 35.2 mm (*p* < 0.001). This corresponds to an average change in limb length of 4.3 ± 2.9 mm, which amounts to 0.652% ± 0.434%. The mean tibial length also increased significantly from 238.3 ± 15.1 mm to 240.2 ± 15.5 mm (*p* < 0.001), but there was no change in femoral length post-operatively (*p* = n.s.). In addition, there was a significant increase in the HKA angle and MPTA from 172.6 ± 0.5° to 180.4 ± 0.4° and from 84.9 ± 0.3° to 91.5 ± 1.1° respectively (*p* < 0.001). The PTS changes from before (9.0 ± 0.5°) to after surgery (9.1 ± 0.5°) were also not statistically significant.

**Table 2:** Pre-operative and post-operative limb lengths, tibial lengths, femoral lengths, HKA angles, MPTA, PTS, planned and actual correction angles

	Pre-HTO	Post-HTO	*p* value
Limb length (mm)	541.2 ± 34.9	545.5 ± 35.2	< 0.001
Tibial length (mm)	238.3 ± 15.1	240.2 ± 15.5	< 0.001
Femoral length (mm)	302.0 ± 19.5	302.0 ± 19.5	0.658
Correction angle (°) [planned vs actual]	8.8 ± 0.6	7.8 ± 0.5	0.041
HKA angle (°)	172.6 ± 0.5	180.4 ± 0.4	< 0.001
MPTA (°)	84.9 ± 0.3	91.5 ± 1.1	< 0.001
PTS (°)	9.0 ± 0.5	9.1 ± 0.5	0.738

*Legend*: *HTO* High Tibial Osteotomy, *HKA* Hip-Knee-Ankle Angle, *MPTA* Medial Proximal Tibial Angle, *PTS* Posterior Tibial Slope

Based on the above results, a further investigation was performed on dichotomous subgroups comparing patients with larger opening widths of > 8 mm versus patients with smaller corrections of less than or equal to 8 mm (Table 3). Thirty-four patients were reported with smaller opening widths  ≤ 8 mm and 57 patients with larger opening widths of  > 8 mm. The change in limb length in patients with smaller opening widths  ≤ 8 mm and larger opening widths of  > 8 mm were 0.578% ± 0.224% and 0.722% ± 0.295% respectively. There was no significant difference in the change in limb length between both groups (*p* = n.s.).

**Table 3:** Change in limb length between patients with opening widths  ≤ 8 mm and  > 8 mm

	Opening width ≤ 8 mm	Opening width > 8 mm
Change in limb length	0.578% ± 0.224%	0.722% ± 0.295%
Number of patients	34	57
***p* value**	n.s	

**Fig. 1:**
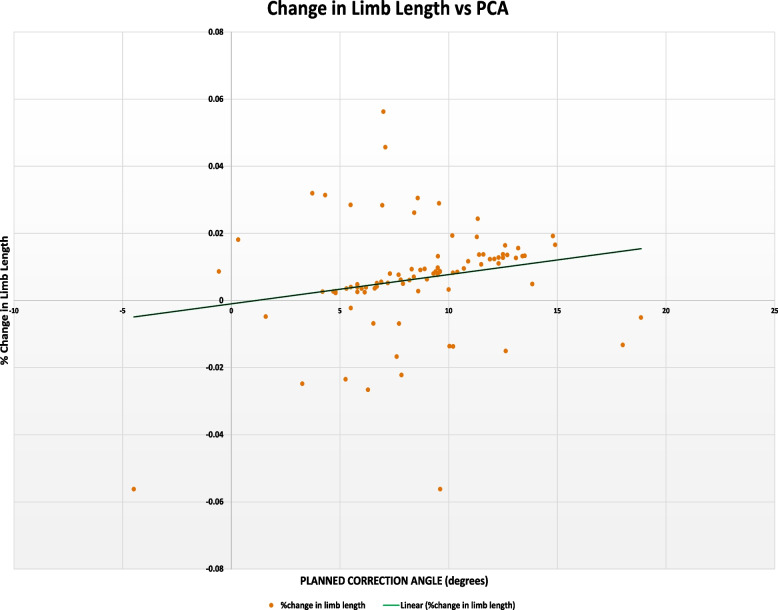
Linear correlation graph of Change in Limb length versus planned correction angle in MOWHTO, which shows weak correlation between % change in limb length versus planned correction angle (*R*.^2^ = 0.196)

**Fig. 2:**
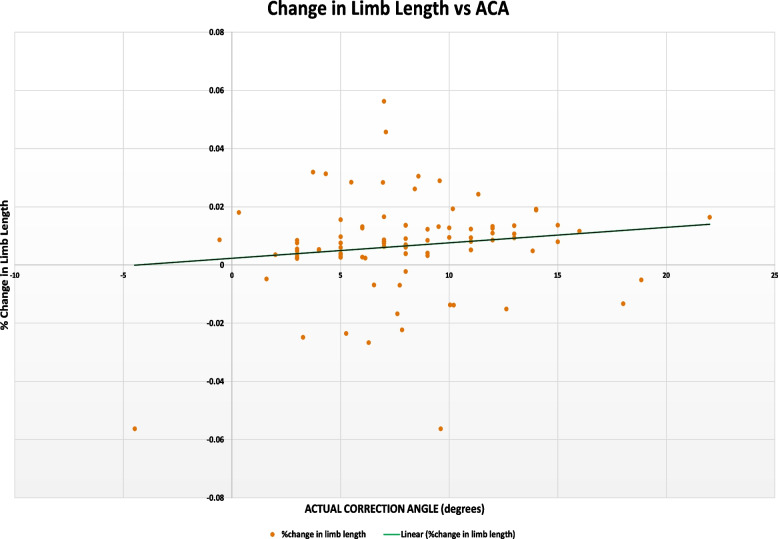
Linear correlation graph of Change in Limb length versus actual correction angle in MOWHTO, which shows weak correlation between % change in limb length versus actual correction angle (*R*.^2^ = 0.140)

The secondary outcomes of the study were to investigate the correlation between planned and actual correction angle versus limb length change. Linear regression was performed to assess the correlation between planned and actual correction angles against change in limb length. There was a very weak positive correlation between limb length change versus planned correction angle (*R*^2^ = 0.196, 95%CI [-0.012, 0.387]) (Fig. 1). Similar observations were made between limb length change verse and actual correction angle (*R*^2^ = 0.140, 95%CI [-0.068, 0.336]) (Fig. 2).

## Discussion

The most important findings in our study were that (i) the increase in limb length following MOWHTO was statistically significant (*p* < 0.05) but not clinically significant (change is  < 10 mm) and (ii) there was no significant difference in the change in limb length between patients with smaller opening widths and larger opening widths.

Our findings corroborate with other studies which show that limb length tends to increase after MOWHTO [5, 12]. This is clinically important as the limb length discrepancy has been shown to be associated with gait deviations and pain [20]. However, it is difficult to determine the magnitude of LLD which is clinically significant. Hinarejos *et al. *[9] and Khamis et al. reviewed and found evidence to support that gait deviations occur starting from a discrepancy of  > 1 cm [11]. In addition, Gordon et al. reviewed studies dealing with the effects of LLD and reported that there is a consensus that LLD  > 2.0 cm often result in pathologies [8]. This means that although this study shows a statistically significant change in limb length after HTO, the clinical significance is debatable given that the change is less than 5 mm. This is encouraging as it enables clinicians to more confidently counsel patients that the likelihood of post-operative gait changes is low. However, this has to be confirmed with further studies that include the functional assessment of patients.

It was also interesting that there was no significant difference in the change in limb length between patients with smaller (≤ 8 mm) versus larger (> 8 mm) opening widths. The lack of a significant LLD when using larger opening widths could be reassuring, but needs to be confirmed with further studies as limb length is a multi-factorial figure, affected by other aspects such as standing posture, pelvic tilt, and scoliosis [13]. A recent study by Ackermann et al.was noted with interest; It indicated that OWHTO results in a statistically significant increase in limb length [1].

Initially, the authors intended to generate a mathematical model based on the results, allowing for patient-specific estimation of limb length change. A mathematical model linking correction angles and change in limb length could improve patient expectations of anatomical results, and contribute to better patient satisfaction post- HTO. This was ultimately not possible as the coefficient of determination (*R*^2^) values were poor (weakly positive). The poor correlation may be due to many other factors, some of which were mentioned above, that can affect limb length—including pelvic tilt, scoliosis, standing posture, and severity of osteoarthritis, to name a few [22]. This makes room for future study expansion where these factors can be assessed and measured and later used in conjunction with the planned and actual correction angles to better predict changes in limb length. If it is deemed that other factors significantly contribute to limb length change, these factors ought to be estimated pre-operatively during surgical planning and if possible, measures should be taken to minimize the post-operative limb length discrepancy.

When translating these results to clinical practice, we observe that while our results are consistent with other studies regarding the statistical significance of limb length change, this does not translate to clinically significant changes in length. Surgeons should thus expect that post-operative MOWHTO patients will have a limb length discrepancy as also demonstrated in other studies. The lack of a statistically significant difference in limb length is reassuring. Patients will be less likely to demonstrate symptoms arising from LLD such as gait disturbance and instability. This is important when surgeons review patient outcomes as they may correlate with poor post-operative patient satisfaction. Conversely, patients with pre-existing LLD secondary to medial compartment arthritis may not demonstrate clinicaaly significant correction after MOWHTO. This plays an important role in the pre-operative counselling of patients and the management of post-operative gait retraining in this group. Even though there is still debate on the LLD required to be considered significant, surgeons undertaking MOWHTO in the Southeast Asian population can counsel patients that the changes are unlikely to be clinically significant or cause a noticeable change in their gait.

Our study has several strengths which includes the correlation of data from 2 independent surgeons and a wide distribution of patients around gender, laterality and ethnicities as representative of the Southeast Asian population. The number of patients used was also in excess of the calculated power required for the study. Results were also correlated between 2 independent observers to reduce inter-observer errors. Some limitations include the poor correlation coefficients and increasing the sample size that could allow for greater reliability of our results. In addition, there is a need for a long-term follow-up for a longitudinal assessment of patient satisfaction, discomfort, pain, gait deviations, and for any pathologies following changes in limb length. Finally, the possibility of non-linear correlation (quadratic, exponential, polynomial etc.) could be considered to predict change in limb length from planned and actual correction angles.

## Conclusion

In conclusion, post-MOWHTO change in limb length is statistically significant, but current literature suggests that the clinical significance of this change (< 10 mm) is minimal. No reliable significant correlation between opening angles and limb lengths were found. With further investigations into the functional impact of a change in limb length, clinicians could reassure MOWHTO patients on the low risk of post-operative limb length discrepancy. Generating a reliable mathematical model that can predict post-MOWHTO limb length change would also be beneficial to clinicians predicting the anatomical outcomes of HTO.

## Abbreviations

MOWHTO: Medial opening wedge high tibial osteotomy
HTO: High tibial osteotomy
HKA: Hip-knee-ankle angle
MPTA: Medial proximal tibial angle
PTS: Posterior tibial slope
BMI: Body mass index
ICC: Intraclass correlation coefficient
CI: Confidence intervals
LLD: Limb length discrepancy
